# How Science Supports Honey Bees: Identification of Research on Best Practices in Beekeeping

**DOI:** 10.3390/insects16101025

**Published:** 2025-10-04

**Authors:** Kristina Gratzer, Veronika Musalkova, Robert Brodschneider

**Affiliations:** Department of Biology, University of Graz, 8010 Graz, Austriarobert.brodschneider@uni-graz.at (R.B.)

**Keywords:** hive management, *Apis mellifera*, *Aethina tumida*, *Varroa destructor*, *Paenibacillus larvae*

## Abstract

Honey bee health and productivity are strongly linked to management practices and biosecurity measures. We collected and analyzed 744 practice records from 191 peer-reviewed field studies published since 1995, each documenting the impact of a specific hive intervention on colony health or productivity parameters. Practices were categorized into good beekeeping practices (n = 128, 17.2%) and biosecurity measures (n = 616, 82.8%) and grouped by management or pathogen theme, geographic region, and season. Most research originated from Europe (34.6%) and North America (33.4%), with nearly all focused on *Apis mellifera* (99.9%). *Varroa* control dominated (57.0%), followed by general apiary management (17.2%) and american foulbrood (9.7%). For varroosis, “soft” acaricides such as oxalic and formic acid accounted for 58.5% of records, while “hard” synthetic products represented 21.0%. Within general apiary management, colony management was most frequent (46.9%). For american foulbrood, antibiotics (41.7%) and biotechnical methods (22.2%) were prominent. *Tropilaelaps* app. treatments relied mainly on “soft” acaricides (81.5%), while small hive beetle control focused on in-hive traps (55.9%). Seasonally, most interventions occurred from August to October, with AFB measures peaking in spring and early summer. The dataset highlights regional research gaps and offers a structured, expandable framework to guide future research and support evidence-based decision-making in beekeeping and advisory services.

## Simple Summary

Honey bees are essential for food production and healthy ecosystems, but they face many threats from pests, diseases, and poor management. Beekeepers around the world use different practices to keep their colonies healthy, yet it is often unclear which methods work best. In this study, we collected and analyzed results from 191 scientific field studies published since 1995. We focused only on research that tested practices directly on honey bee colonies under real beekeeping conditions. Altogether, we found 744 different practice records and evaluated their effects on colony health and productivity. Most studies dealt with controlling the *Varroa* mite, one of the most damaging pests of honey bees. Other important topics were the management of colonies, the prevention of american foulbrood disease, and the control of other parasites and pests. We also looked at when during the year the practices were applied and in which regions of the world. The results show that there is no single solution, but a variety of methods can help improve colony health. This study provides a practical framework that can guide beekeepers, advisors, and policymakers in making evidence-based decisions to support sustainable apiculture for beekeepers, veterinarians, and extension professionals.

## 1. Introduction

Health and productivity of livestock depend on management practices and biosecurity measures. Good beekeeping management keeps livestock protected from major stressors, reduces the risk of diseases, and positively influences the productivity and economic profit of the farmer [1,2]. Managed honey bees as livestock are housed in artificial structures and subject to human selection, yet they forage freely in the landscape. According to the World Organisation for Animal Health (OIE), bees are classified as terrestrial animals and thus fall under veterinary care [3]. Honey bees are the most economically important pollinators globally, significantly contributing to agricultural productivity and ecosystem stability [4]. In addition, bees provide a wide variety of products: honey, pollen, propolis, wax, royal jelly, and venom. Beekeepers also sell colonies and rear queens, further diversifying their economic activities [1].

Large-scale honey bee colony losses have been reported worldwide. Numerous surveys and research projects have sought to identify the primary drivers of these losses [5,6,7,8,9]. These studies often highlight the *Varroa* mite (*Varroa destructor*) as the major threat to *A. mellifera* colonies. Originating on *Apis cerana*, *V. destructor* exploits the lack of a natural host–parasite balance, leading to colony collapse within a couple of years if untreated. This parasite compromises bee health by weakening immune responses [10,11], transmitting viruses [12,13], and impairing physiological development [14,15].

Bacterial infections remain a major contributor to honey bee colony losses worldwide. Among them, american Foulbrood (AFB), caused by the spore-forming *Paenibacillus larvae*, and european foulbrood (EFB), caused by *Melissococcus plutonius*, are particularly relevant due to their persistence and ease of transmission [16,17]. Further, EFB infections are often complemented by secondary pathogens such as *Paenibacillus alvei*, *Brevibacillus laterosporus,* and *Enterococcus faecalis* [16]. Beyond bacterial pathogens, parasitic mites also pose threats to honey bees. *Tropilaelaps* mites (*Tropilaelaps* spp.), originally parasitic on *Apis dorsata*, represent another significant threat, particularly in Asia. They outcompete *Varroa* mites under certain conditions due to their shorter phoretic stage and rapid reproduction [18,19,20]. Infestations can lead to brood mortality and colony decline [21]. Continuous monitoring is therefore essential, as highlighted by ref. [22], who introduced the “rapid brood decapping” method to assess *T. mercedesae* infestation in brood. While this work does not provide direct evidence of westward spread into Europe, it underscores the importance of surveillance tools to track potential range expansions. The small hive beetle (*Aethina tumida*), an invasive species originating from Africa, has also established itself in several parts of the world, including the Americas, Australia, and more recently in Europe [23,24]. It damages colonies by consuming brood, pollen, and honey, and its presence can ultimately trigger colony collapse [25].

Such biological threats gain further relevance in the context of globally practiced beekeeping and the diverse environmental conditions in which honey bees are now kept. Originally limited to the Old World, honey bees have since been introduced into a wide range of climates and landscapes by humans. The primary species in apiculture is the Western honey bee *A. mellifera*, followed by the Eastern honey bee *A. cerana*; they have adapted to diverse environmental conditions, demanding region-specific beekeeping practices [26,27]. Comprehensive measures for prevention, early detection, treatment, and pest control are essential for protecting colony health and ensuring the long-term sustainability of apiculture [3].

This study aims to identify beekeeping and biosecurity practices with demonstrated effects on colony health and productivity. To achieve this, we analyzed peer-reviewed field studies that included comparisons between treatment and control groups under realistic on-field conditions. Practices were categorized into good beekeeping practices, referring to regular hive management actions such as timely feeding, queen replacement, or hygienic comb renewal, and biosecurity measures, which focus on preventing the introduction and spread of pathogens within and between apiaries, including quarantine protocols or disinfection routines [1,3]. The impact of each practice on colony health and productivity was evaluated to identify practical recommendations for beekeepers, veterinarians, and advisory services. Results are presented in both tabular and graphical form and are contextualized by region and season.

Although numerous studies on honey bee health exist [28,29,30], comprehensive syntheses that translate these findings into actionable, field-tested management recommendations for stakeholders remain limited. Reasons for this include inconsistent study outcomes, differences in regulations, and region-specific applicability. For example, the path from pathogen identification in the laboratory to the availability of effective treatment options for beekeepers is often long and uncertain. This work is intended as a framework for the structured analysis of applied honey bee research and is designed to be flexible enough for future expansion as new studies become available. The categorization is based on the lists of practices compiled and prioritized by [1,3].

## 2. Materials and Methods

To ensure practical relevance and comparability, we focused on studies in which the subject unit was a honey bee colony, the research was conducted under field conditions, and both experimental and control groups were included. Only publications from 1995 onwards in English language were considered. By applying these criteria, we established a structured framework that synthesizes existing applied research and is designed to be continuously expandable as new studies become available.

All scientific publications were obtained through the Google Scholar search engine. Out of 341 reviewed papers, 191 were included in this study, considering a total of 12,400 beehives used for their field studies. Papers representing reviews or survey data were excluded; however, these were sometimes useful for obtaining an overview of the research conducted so far on the related theme. Further excluding criteria cover papers based on laboratory experiments or studies where a single honey bee or a group of bees in cages represented the studied object.

Our next step was to compile all relevant data into a MS Excel spreadsheet (Microsoft Corporation, Redmond, WA, USA), which served as the basis for the summary analyses. This spreadsheet, referred to as Supplemental Material S1, represents the core output of this work. Each reviewed publication was systematically screened for relevant practices. In the resulting table, each row corresponds to a single practice record, defined as one documented application of a specific practice and its observed effect. A single publication may contain multiple practices, or a single practice evaluated for multiple outcomes, each of which is represented as a separate practice record. For example, caging the queen may reduce *Varroa* mite infestation levels (one row), while also affecting honey yields (a second row). Throughout this study, we therefore use the term practice record to describe these individual entries. To ensure clarity and consistency in terminology, practice refers to a specific beekeeping measure or management approach investigated in a study, while practice record denotes its application and evaluation in a particular experimental context. The term sub-theme is used to describe categories within broader thematic groups (e.g., “soft acaricides” within the theme varroosis). When referring specifically to interventions against pests or pathogens, particularly in the context of varroosis, *Tropilaelaps* spp., or american foulbrood, we use the term treatment.

To allow for comparability across studies with varying methodologies, each practice record was described as precisely as possible while maintaining a simplified, standardized format. For example, numerous methods have been investigated in research in an effort to combat varroosis. Additional technical details such as mode of application or substance concentration were included in separate columns where available, whereas more specific information on the practice record must be retrieved from the original paper. As the practices were identified, these were subsequently categorized into eight thematic areas: general apiary management, small hive beetle infestation (*Aethina tumida*), american foulbrood (AFB), chalkbrood, european foulbrood (EFB), greater wax moth (*Galleria mellonella*), *Tropilaelaps* spp. infestation, and *Varroa* mite infestation [1,3] (Supplemental Material S1). For the sub-themes AFB and varroosis, we conducted additional analyses. Specifically, we examined the seasonality of practice application for both sub-themes. For varroosis, we also assessed the efficacy of practices, with a particular focus on the most frequently studied practice type: treatments involving oxalic acid.

For the category of “biosecurity measures”, the impact was further categorized to provide a more precise classification of significant efficacy. Positive effects were subdivided into three levels: “positive” for efficacy greater than 85%, “moderate positive” for efficacy between 50% and 85%, and “low positive” for efficacy below 50%. Measures with less than 10% efficacy were classified as having “no effect”. Efficacy data, means and numbers of application, concentration, length of the procedure and dosage are provided in separate columns. If unavailable, the classification from the research paper is used. Unless otherwise stated, no additional statistical analysis was conducted, and effect sizes or significance levels were not recalculated. Therefore, the frequency values presented in this work reflect how often certain practices were associated with specific outcomes in the reviewed studies, rather than indicating the strength or statistical significance of those effects. As such, results should be interpreted as descriptive evidence of reported associations, not as quantified effect estimates.

For the geographic classification we used a simplified subdivision (“continent”, “country”, “region”) with the main emphasis put on the division of European regions. “Continent” includes Europe, North America, Central America, South America, Asia, Africa, Oceania; “region” is only used for European and includes division into six regions: Northern, Western, Central, Southern, Eastern and South-eastern Europe.

Regarding the seasonal data, we recorded these in the Supplemental Material S1 by month. To ensure consistency between hemispheres, seasonal data from the Southern Hemisphere were adjusted by shifting six months forward, aligning July–December with January–June of the Northern Hemisphere. The column “species” relates to the biological classification of the investigated honey bee colonies.

Additionally, we also recorded the year of publication and year of the experiment, number of colonies involved in the research and the reference as well. When necessary, we also added a comment in a separate column. For visualization, the software GraphPad Prism 8.0.2. (GraphPad Software, San Diego, CA, USA) was used.

## 3. Results

### 3.1. Geographic and Temporal Distribution

**Figure 1:**
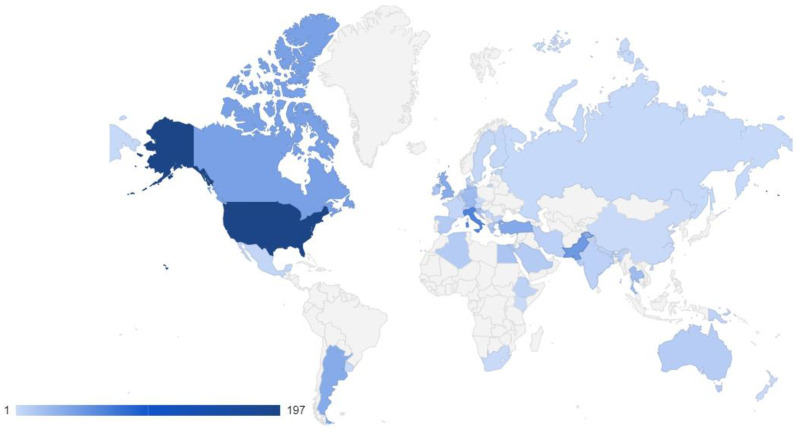
World map showing the density of identified practices. The density of the color increases with the number of identified practice records; light blue represents fewer, dark blue represents more practice records.

Most research in our study originates from Europe (34.6%) and North America (33.4%), followed by Asia (19.5%), Africa (5.1%), South America (5.0%), and Oceania (2.4%). Research spans 42 countries (Figure 1), with the USA leading honey bee health studies (26.5%). In Europe, key contributors include Italy (8.9%), the UK (4.7%), Germany (3.2%), and Slovenia (3.1%).

**Figure 2:**
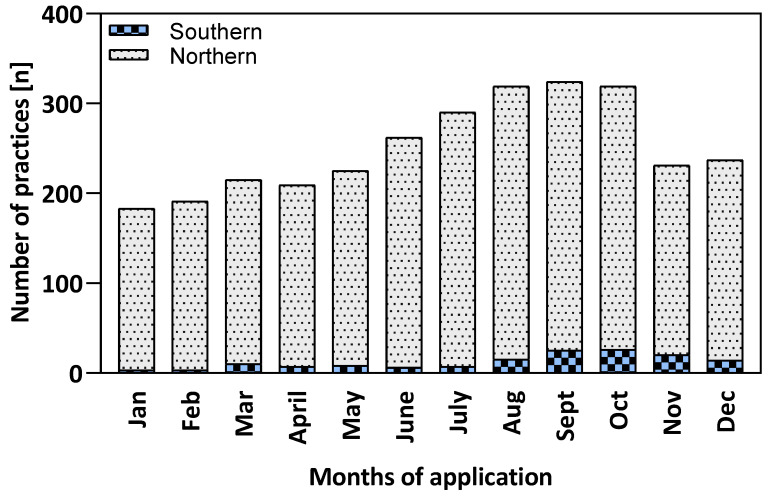
Monthly distribution of identified beekeeping practice records by region. Bars are stacked to show contributions from Northern and Southern hemisphere to the global total (n = 744). Most practice records were documented in the Northern regions.

To better understand temporal patterns in the implementation of beekeeping practices, we analyzed the seasonality of application across all practice records with available monthly data (Figure 2). We shifted the seasonal data of the Southern Hemisphere at 6 months rate to get a unified calendar (see Section 2). After shifting, globally, the number of practice records peaks between July and October, with a noticeable decline in the winter months forming a seasonal pattern. Since the numbers for the Northern Hemisphere always account for 90% up to 98% of the global numbers, the observed pattern is largely similar. This is probably caused by the high proportion of studies on *Varroa* in the literature. In contrast, the Southern Hemisphere shows a smaller but distinct peak during the months of September to November.

### 3.2. Practice Themes and Frequencies

We identified 191 studies resulting in a total of 744 practice records, capturing distinct combinations of practices and outcomes. Practice records were categorized into 8 themes, which are summarized in the Supplemental Material S1. Biosecurity measures accounted for the majority of these practice records (n = 616, 82.8%), while records under good beekeeping practices represented a smaller portion (n = 128, 17.2%). The theme of varroosis was by far the most extensively covered, with 424 practice records (57.0%) addressing this critical issue. General apiary management followed with 128 practice records (17.2%), reflecting its importance in overall colony health and productivity. American foulbrood (AFB) was the third most represented theme with 72 practice records (9.7%), highlighting its continued relevance as a significant bacterial disease in apiculture. Other notable themes included *Tropilaelaps* mite infestation (65 practices, 8.7%) and small hive beetle (aethinosis, 34 practice records, 4.6%). Lesser-studied themes such as european foulbrood (EFB), greater wax moth, and chalkbrood collectively accounted for fewer than 3% of the total practice records, with only 19, 1, and 1 practice records, respectively.

For example, colony management was the most frequent sub-theme (46.9%) for general apiary management. Recent research on *Varroa* mites (varroosis) focuses on “soft” acaricides accounting for 58.5% of practice records, followed by “hard” acaricides (21.0%). With 41.7% antibiotics remain the most important method to combat AFB, followed by 22.2% of biotechnical methods. Studies on treating *Tropilaelaps* spp. focus mainly on “soft” acaricides (81.5%), with biotechnical methods (7.7%) and “hard” acaricides (9.2%) being less common. For the small hive beetle (*Aethina tumida*), in-hive traps dominate (55.9%), followed by hive entrance modifications (17.6%). Table 1 provides a detailed description of the sub-themes and their distribution for general apiary management, varroosis, AFB, tropilaelapsis and aethinosis.

### 3.3. General Apiary Management

The most abundant sub-theme was colony management (n = 60). Practice records associated with reduced annual or winter losses include starting new colonies (splits: 11.7%; nucleus colonies: 11.7%), specific hive types (small hives: 10.0%; winter isolation: 3.3%; summer isolation: 1.7%), and swarm control (8.3%). The operational approach, whether organic, conventional, or chemical-free, also plays a significant role, with each type equally represented (6.7%) in the data. Additionally, comb management (13.0%) and the use of domestic queens (8.7%) show positive effects on colony survival. All nine practice records linked to increased honey yields are evenly represented (11.1% each) and are primarily related to colony management strategies (Supplemental Material S1).

According to some researchers, infestation levels (*Varroa*, viruses, *Nosema*) have been positively influenced by the method of starting new colonies (splits 12.0%, nucleus colonies 12.0%), comb hygiene (12.0%), swarming (8.0%), operational type (conventional 8.0%, organic 8.0%), natural forage (8.0%), and less so by annual queen replacement (4.0%) and pollen supplementation (4.0%; Table 2).

Research on queen management primarily focuses on annual queen replacement (42.9%) and its effects on colony losses, productivity, and health. Additional studies compare the performance of domestic (21.4%) versus imported (28.6%) queens (Supplemental Material S1).

Nutrition of the colonies can be influenced by location of an apiary within different landscape contexts, for example, whether it is situated near natural habitats, agricultural fields, or urban areas. These investigations represent 24.4% and 17.8% for natural and agricultural forage, respectively. Supplemental feeding can mean both: providing bees with additional protein or energy sources. Protein sources may be natural pollen (represented with 17.8%), protein substitute (with 13.3%) or amino acid product (4.4%). As a source of energy, traditionally sugar syrup (11.1%) or high fructose corn syrup (11.1%) can be fed to honey bees.

Four practices were linked to higher annual or winter colony losses: chemical-free operations (20.0%), imported queens (40.0%), long-distance transportation (20.0%), and protein supplementation (20.0%). Additionally, lower honey yields were reported for chemical-free operations (50.0%) and shook swarms (50.0%, Supplemental Material S1).

### 3.4. Varroosis

Varroosis is of main interest in our study, with 424 identified practice records derived from 114 papers. The most extensively researched are “soft” acaricides (n = 248), particularly oxalic acid (36.3%), formic acid (27.8%), and thymol (22.2%). Studies on “hard” acaricides (n = 89) primarily examine tau-fluvalinate (30.3%), amitraz (28.1%), coumaphos (23.6%), and flumethrin (18.0%). Biotechnical methods for *Varroa* control include drone brood removal (22.0%) and hyperthermia (22.0%), queen caging (17.1%), powdered sugar dusting, requeening, and small cell foundation (7.3% each), alongside swarming and total brood removal (4.9% each).

Among the 177 practice records with a positive effect on *Varroa* reduction, oxalic acid was most frequently mentioned (21.5%), followed by other “soft” acaricides such as formic acid (10.7%) and thymol (9.6%). Common “hard” acaricides, including tau-fluvalinate, coumaphos, and amitraz, also proved effective. Biotechnical methods like drone brood removal, queen caging, hyperthermia, and trapping combs were additionally reported. Practices like “queen caging + oxalic acid” and regular mite monitoring were positively associated with both increased honey yields and reduced colony losses (Table 3).

Adverse effects (n = 24) on honey bee colonies have been reported associated with the use of formic acid (37.5%), oxalic acid (29.2%), hyperthermia (12.5%), thymol (12.5%), queen caging (4.2%) and VarroMed (4.2%). Some substances have been reported to leave residues in wax and/or honey (n = 6), namely coumaphos, tau-fluvalinate and thymol. Reduced honey yields (n = 3) have anecdotally been observed with queen caging, hyperthermia and swarming. This highlights the importance of balancing efficacy with potential drawbacks in *Varroa* management strategies.

**Figure 3:**
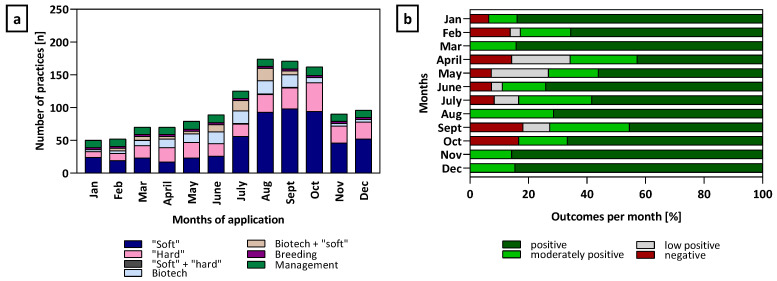
Practices used to treat varroosis. (**a**) categories of *Varroa* mite treatments over the months of application: “soft”, “hard” or a combination of “soft” and “hard” acaricides, biotechnical methods or in combination with “soft acaricides, breeding for resistance and colony management practice records. (**b**) relative monthly outcomes of practice records including oxalic acid treatments for *Varroa* control. Bars represent the percentage share of each efficacy outcome category per month (negative, low positive, moderately positive, positive efficacy). n = 424 practice records.

The seasonal distribution and effectiveness of various practices used to treat varroosis show that the majority of treatments include the use of “soft” acaricides, which were mainly used between July and October, aligning with the period of highest *Varroa* pressure after honey harvest. Biotechnical methods and management strategies are used more evenly throughout the year but increase slightly in late summer (Figure 3a). Significant high treatment success (“positive”) of oxalic acid is within a range of 84.6% to 85.7% efficacy most common during broodless periods (winter in the Northern hemisphere). In contrast, treatments applied in early spring (March–May) tend to result in a more varied range of “positive” outcomes between 42.8% to 84.2%, including a higher share of moderate, low positive and even “negative” (7.3–14.3%) effects. This trend is similar for the summer months (June to August) with a maximum of 74.1% “positive” and 8.3% “negative” outcome in June and July, respectively (Figure 3b).

### 3.5. American Foulbrood

The most research on AFB has been evaluating the use of antibiotics (n = 30). Of these, tylosin has been evaluated most often (53.3%), followed by oxytetracycline (33.3%). Erythromycin and lincomycin was evaluated in a small number of studies.

Practices for treating AFB include shook swarms, primarily for spore reduction (69.2%) and colony recovery (29.1%). Antibiotics such as tylosin and oxytetracycline positively impact the clinical course of the disease but may leave residues in honey. Additional practice records with positive or negative effects related to AFB are summarized in Table 4.

**Figure 4:**
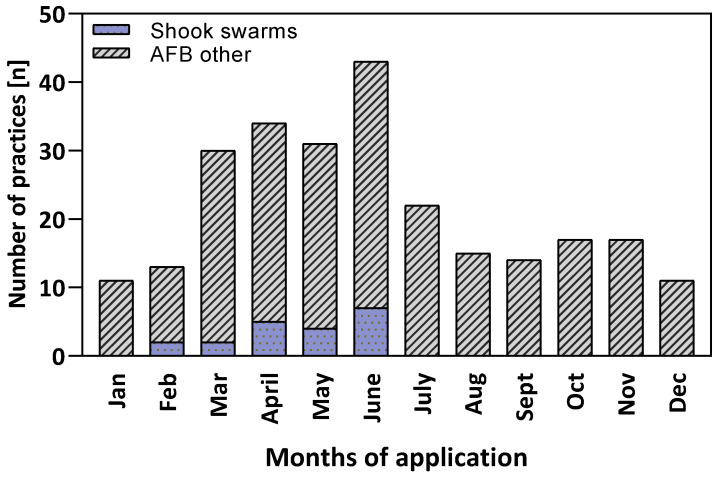
Monthly distribution of practice records targeting american foulbrood (AFB), including shook swarm applications and others. Bars represent the total number of AFB-related practice records per month, with the subset of shook swarm treatments shown in blue (n = 74).

The seasonal distribution of practice records targeting american foulbrood (AFB) reveals a clear peak during spring and early summer, with the highest number of 29 and 36 interventions recorded in April and June, respectively (Figure 4). Among these, the shook swarm method stands out as a targeted strategy used exclusively during the period between February and early summer in June with a minimum of 2 and a maximum of 7 counted practice records, respectively.

### 3.6. Small Hive Beetle

Two sub-themes dominate as treatment against SHB infestation: in-hive traps (n = 19) and hive entrance modification (n = 6). In-hive traps can incorporate chemical substances such as fipronil (47.4%), coumaphos (15.8%), acetamiprid (5.3%) or slaked lime (5.3%) or a bait (5.3%) or can be constructed as a pitfall (10.5%). Hive entrance can be modified by its upper location (33.3%), by narrowing the entrance to the size of a small pipe (33.3%) or by combination of these two methods (33.3%).

In general, practice records on aethinosis were rather underrepresented 4.6% of 744 records. Positive effect on SHB infestation reduction has been described with the use of in-hive traps with fipronil (36.4%). Further, traps with acetamiprid or coumaphos, baited traps, hive management, hygienic behavior, traps in front of the hive and hive modification have occasionally been evaluated with positive effect on SHB reduction, formic acid, oxalic acid and upper location of the hive entrance seem to have no or little effect on the SHB infestation of the honey bee colonies.

### 3.7. Tropilaelaps spp.

Practice records on *Tropilaelaps* infestations were rather underrepresented with 8.7% of 744 practices, respectively. A detailed list of treatments is found in the Supplemental Table S1.

Similarly to the *Varroa* mite, “soft” acaricides, “hard” acaricides and biotechnical methods are also used to investigate the control of the *Tropilaelaps* mite. Most research is assigned to the sub-theme “soft” acaricides (n = 53) and among these, formic acid (30.2%), thymol (13.2%) and sulfur (11.3%) have been evaluated. Hop acids and essential oils were occasionally studied as well. Classical synthetic varroacides such as flumethrine, amitraz and coumaphos, have rarely been investigated as countermeasures for *Tropilaelaps* spp. (n = 6), as well as some biotechnical methods such as queen caging, queen removal, splitting colonies or powdered sugar dusting. Effective and ineffective treatments for the *Tropilaelaps* mite include formic acid (35.3%), flumethrine (11.8%) and sulfur (11.8%). Insufficient reduction has been briefly observed when investigating hop acids (50.0%), amitraz (25.0%) and sulfur (25.0%).

### 3.8. Species and Subspecies Used

With the exception of one study, all publications investigated honey bee colonies of the species *Apis mellifera* (99.9%). In most cases (73.7%), the subspecies was not further specified. Among those that did provide more detail, *A. m. ligustica* (12.2%) and *A. m. carnica* (6.3%) were the most commonly used. Only a single study (0.1%) was conducted on *Apis cerana cerana* Fabricius, indicating that this species is rarely used in applied field studies worldwide. The remaining records referred either to specific subspecies such as *A. m. intermissa*, *A. m. mellifera*, or hybrid lines like Buckfast, or involved studies where more than one subspecies was used.

## 4. Discussion

This study presents a structured synthesis of 744 beekeeping practice records from 191 peer-reviewed field studies and offers a comparative framework to assess the efficacy, seasonality, and geographic relevance of these practice records. The intention is to provide a growing foundation for applied apicultural research, targeting beekeepers, extension services, representing people who link research and practice, and policymakers seeking reliable, field-tested strategies to support honey bee health and productivity.

Identifying globally best practices remains difficult due to the strong influence of environmental and climatic conditions, regulatory variations by local governments, regional differences in beekeeping practices, and varying beekeeping philosophies. These factors limit the comparability of practices and outcomes across studies. Despite efforts to achieve comprehensive coverage, manual screening and methodological heterogeneity among studies may have introduced limitations or selection biases. To improve reproducibility and reduce potential selection bias, we recommend applying standardized systematic review methodologies [30,31].

Our study focused on major health-related drivers, namely *Varroa destructor*, american foulbrood (AFB), and general apiary management, while also covering underrepresented but emerging themes such as *Tropilaelaps* spp. and small hive beetle (*Aethina tumida*). We collected scientific data from 42 countries and all continents inhabited by honey bees. The majority of studies included originate from Europe and North America, followed by Asia, Africa, South America and Oceania and the main study object was *A. mellifera* L. Despite broad geographic coverage, practices from the global South remain underrepresented, suggesting the need for more field-based studies that reflect diverse ecological and socioeconomic contexts. In addition, all practice records were categorized by season. *Varroa* treatments were most frequently applied in August and September following honey harvest, while interventions against AFB were concentrated in spring and early summer, during periods of intense brood rearing. Across all themes, most practices were implemented between August and October. The resulting dataset (Supplemental Table S1) provides a foundation for more targeted analyses. Focused, topic-specific reviews, for example, on biotechnical *Varroa* control or seasonal timing of AFB interventions, could build on this work. Here, we present only a few illustrative examples in the form of simple calendars.

Good beekeeping practice (GBP) plays a central role in sustaining profitable and healthy colonies [2]. Furthermore, it also prevents or significantly reduces the impact of honey bee diseases. We identified 128 practice records from 27 peer-reviewed studies in the categories, colony management, queen management, feeding and watering, and hygiene [1].

Several studies had a particularly strong influence on our findings, identifying best management practices (BMPs) that significantly reduced colony losses, pathogen levels and increased profitability [2,32]. Building on this, ref. [33] provided updated survey data on colony losses in the USA, offering critical baseline information to contextualize management practices and their outcomes. Further, ref. [9] developed a tool to identify high-impact practices through data-driven analysis.

Our findings also support the ongoing shift toward organic and sustainable beekeeping, although evidence suggests that abandoning treatments, especially for *Varroa*, altogether may increase colony losses [34]. Instead, the effective use of soft acaricides and biotechnical methods can achieve comparable outcomes, if colony management is adapted to seasonal and local contexts.

The investigations involved in our study uniformly confirm the positive influence of low colony densities within the apiary on the *Varroa* mite infestation level and the colony mortality [35,36]. Further, overwintering, performance and health of the honey bee colonies can be significantly impacted by certain hive modifications, in accordance with the climatic region, for instance in hot summers in arid regions vs. harsh winters in the northern countries [26,27]. Recent findings support such adaptions. Thus, hive insulation with simple covers can reduce food consumption and improve overwintering survival, even in moderate climates [37]. Swarming usually results in economic losses for the beekeeper but can provide natural brood interruption, helping reduce *Varroa* levels. As an alternative, the technique of splitting colonies takes advantage of brood interruption without the negative effects of swarming [38,39].

Queen management is a critical component of colony performance. Frequent queen losses are a known risk factor in the annual losses’ statistics [40,41]. Hence proper queen management has been thoroughly studied. Ref. [42] further demonstrated that preparation of queenless colonies for at least five days significantly improves virgin queen acceptance. If the queen replacement was performed with hives being queenless for only one day, the acceptance rate of immature queens was significantly lower. Our findings support existing evidence that younger queens, locally bred queens, and proper requeening timing improve colony outcomes [7,43,44,45,46]. Such data-driven recommendations complement our broader results and emphasize the need to adjust requeening strategies to colony condition and timing. Established breeding programs allow to increase the honey production and positively modify the behavior of the honey bee colony.

While mating behavior is recognized as important, it remains understudied in current research. Queens mate with multiple drones, enhancing genetic diversity and resilience, brood viability, and disease resistance [47,48]. This underlines the importance of using well-mated queens and ensuring good mating conditions during rearing. Additionally, recent findings show that genetical resistance traits, such as mite non-reproduction, can be maintained through selective breeding under natural mite pressure [49]. While still emerging, these approaches hold promise for reducing chemical dependency to reduce chemical treatments and improve colony health via genetics-based management [50].

Nutrition operates as a central determinant of colony performance and resilience, mediating interactions between landscape, season, disease susceptibility, and productivity. Studies in our dataset show that pollen diversity, floral richness, and targeted supplemental feeding can significantly enhance immunity, brood development, and overwintering success. Thus, a diverse diet supports colony health [51,52,53]. However, findings on protein supplements remain rather inconsistent.

As shown in an analysis of 100 years of honey bee research, *Varroa*-related studies became a central focus in the 1990s and remain highly relevant today [30]. Several in-depth reviews have been written that provide a thorough summary of the biology and control of the parasitic mite [14,15,54,55,56]. We identified 424 practice records from 114 original articles dedicated to *Varroa* control, covering chemical, biotechnical, and integrated pest management (IPM). No universal solution emerged, but multiple effective strategies are available.

Recent trends emphasize Integrated Pest Management (IPM) and reduced reliance on “hard” acaricides, aligning with One Health principles that consider animal, human, and environmental health [1]. Our analysis shows that, under appropriate conditions, biotechnical methods and “soft” acaricides, such as oxalic or formic acid, can be as effective as synthetic treatments. Recent reviews [55,56] emphasize the importance of timing and synergy: combining brood interruption with oxalic acid sublimation often yields best results [57]. Nonetheless, synthetic acaricides continue to play a key role. Depending on the region and infestation level, they remain highly effective and usually do not impair colony development. However, resistance issues and residue accumulation must be considered. Coumaphos seems to be less effective, probably due to the development of mite resistance. Additionally, coumaphos leaves the most residues in wax and to some degree also in honey [58,59].

Among synthetic acaricides, amitraz remains most commonly used in Europe [60].

Soft acaricides (e.g., oxalic and formic acid) are promising but require precise application depending on brood presence, temperature, and colony size. Sublimation has been occasionally referred to as the most bee-friendly application method of oxalic acid application. Consistent findings across studies confirm that oxalic acid treatment during the broodless period is highly effective, regardless of application method [57,61,62,63,64,65]. Formic acid and thymol are more sensitive to temperature and colony status, complicating general recommendations. Formic acid may cause negative effects like brood loss or queen damage under suboptimal conditions [66,67,68].

Biotechnical methods vary in efficacy. Brood removal, queen caging, and trapping combs are generally effective [69,70,71], while sugar dusting and small-cell foundations show limited success [72,73]. Monitoring of mite drop post-treatment is critical to evaluate success, despite treatment method [74].

We identified 72 practice records from 21 studies addressing AFB, categorized into several sub-themes. Although antibiotic use is prohibited in some regions, it remains common elsewhere. Correspondingly, many investigations have been performed on efficacy, dosage and application methods of antibiotics. Traditionally, oxytetracyclines has been widely used, which resulted in resistance. Therefore, new antibiotics, such as tylosin or lincomycin have been tested, with tylosin showing the highest efficacy [75,76,77]. However, antibiotic residues in honey remain a concern, which can be supported by our results.

To avoid the use of antibiotics, other approaches to combat AFB have been studied. Traditionally, the stamping-out method, including the burning of infected colonies, remains effective but is accompanied by colony and therefore economic losses. The shook swarm method offers a less destructive, yet effective control measure [78,79]. Strategic long-term monitoring and elimination programs have been proven to be successful in fighting AFB [80]. New innovative and still developing approaches may represent the oral vaccination of honey bee queens [81]. The research on European foulbrood is not as extensive. Three methods of combating the pest have been investigated: antibiotics, biotechnical methods, which technically means the shook swarm method and their combination [82,83].

*Tropilaelaps* spp. mites are a serious threat to *A. mellifera* colonies, particularly in Asia. Compared to *Varroa*, the scientific coverage is limited. The most effective treatment identified in the literature is formic acid evaporation [84,85]. Traditionally, sulfur has been used with reasonable efficacy. Synthetic acaricides may offer additional options, but few studies have evaluated their effectiveness.

Further research is essential to establish effective control strategies for aethinosis. The small list of identified practices reveals that the most promising approach is the use of in-hive traps containing fipronil [86,87]. However, the available studies are scarce and do not cover alternative methods such as external traps, often due to discrepancies between the study design and the criteria for inclusion in this review. As many regions are still SHB-free, preventive strategies were not part of this review but important for further investigation.

## 5. Conclusions

Taken together, the reviewed practices highlight the necessity of an integrated, multi-dimensional approach to honey bee colony management. No single intervention is sufficient on its own; rather, effective beekeeping relies on the interplay of multiple, evidence-based strategies. Practices such as regular *Varroa* monitoring and timely treatments, maintaining high-quality and locally adapted queens, adjusting nutritional support to seasonal forage availability, and implementing hygiene and biosecurity protocols form a cohesive management system. These elements reinforce each other; for instance, brood interruption not only aids in *Varroa* control but also aligns with queen replacement strategies, while diversified forage and supplemental feeding can strengthen colonies’ resilience against both pathogens and environmental stressors. Our results support the development of a practical framework in which colony health is managed holistically, and it is put in context with season, geography, colony condition, and regulatory environment.

This study provides a structured overview of 744 beekeeping practices from 191 systematically selected field studies, each linked to either a positive or negative impact on colony health or productivity. While the dataset is extensive, we acknowledge that certain gaps remain due to database limitations, inclusion criteria, or underrepresented themes. Still, this framework offers a valuable tool for synthesizing applied honey bee research and for prioritizing practices that have been demonstrated to be successful in real-life beekeeping. Future work should build on this foundation by expanding to additional themes, incorporating long-term and region-specific outcomes, and refining recommendations through ongoing international collaboration. The aim of this work is to promote honey bee health, sustainable beekeeping, and the pollination services essential to ecosystems and agriculture. Therefore, we encourage further validation, contribution, and use of this framework and we support evidence-based decision-making for beekeepers, researchers, veterinarians, and policymakers.

## Figures and Tables

**Table 1:** Sub-themes ranked by the percentage representation of identified practice records (n = total number of practice records).

General Apiary Management		Varroosis		AFB *		Tropilaelapsis *		Aethinosis *	
n = 128		n = 424		n = 72		n = 65		n = 34	
Colony management	46.9%	“Soft” acaricides	58.5%	Antibiotics	41.7%	“Soft” acaricides	81.5%	In-hive traps	55.9%
Feeding and Watering	35.2%	“Hard” acaricides	21.0%	Biotechnical methods	22.2%	“Hard” acaricides	9.2%	Hive entrance modification	17.6%
Queen management	10.9%	Biotechnical methods	9.7%	Organic agents	12.5%	Biotechnical methods	7.7%	“Soft” acaricides	8.8%
Hygiene	7.0%	Biotechnical methods + “soft” acaricides	6.6%	Laboratory testing	6.9%	Breeding for resistance	1.5%	Colony management	8.8%
		Colony management	3.3%	Colony management	4.2%			Traps outside of hives	5.9%
		Breeding for resistance	0.7%	Breeding for resistance	4.2%			Breeding for resistance	2.9%
		“Soft” acaricides + “hard” acaricides	0.2%	Stamping-out	2.8%				
				Vaccination	2.8%				
				Biotechnical methods + antibiotics	2.8%				

* Total % do not add up to 100% due to rounding to one decimal place.

**Table 2:** General apiary management: the significant positive impacts of practice records on honey bees. n = total number of practice records.

*Varroa* + Viruses + *Nosema* Infestation		Honey Yields *		Annual/Winter Losses + Number of Colonies *	
n = 25		n = 9		n = 23	
Freezing old brood comb before reuse	12.0%	Annual queen replacement	11.1%	Freezing old brood comb before reuse	13.0%
Starting new colonies by buying nuclei	12.0%	Conventional operation	11.1%	Starting new colonies by buying nuclei	13.0%
Starting new colonies from splits	12.0%	Freezing old brood comb before reuse	11.1%	Starting new colonies from splits	13.0%
Conventional operation	8.0%	Isolated hives in summer	11.1%	Domestic queens	8.7%
Low colony densities	8.0%	Low colony densities	11.1%	Reusing equipment from dead colonies immediately	8.7%
Natural forage	8.0%	Organic operation	11.1%	Annual queen replacement	4.4%
Organic operation	8.0%	Reusing equipment from dead colonies immediately	11.1%	Conventional operation	4.4%
Small hives	8.0%	Starting new colonies by buying nuclei	11.1%	Low colony densities	4.4%
Swarming	8.0%	Starting new colonies from splits	11.1%	Natural forage	4.4%
Annual queen replacement	4.0%			Organic operation	4.4%
Pollen supplementation	4.0%			Pollen supplementation	4.4%
Supplement BEEWELL AminoPlus	4.0%			Small hives	4.4%
Swarming + low colony densities	4.0%			Swarming	4.4%
				Swarming + low colony densities	4.4%
				Wintering strong colonies	4.4%

* Total % do not add up to 100% due to rounding to one decimal place.

**Table 3:** Varroosis: the positive impact of practice records on the *Varroa* mite infestation, honey yields and colony losses. Practice records with frequencies below 1% were summarized as “other”. n = number of practice records.

*Varroa* Infestation Levels		Honey Yields *		Colony Losses *	
n = 177		n = 7		n = 15	
Oxalic acid	21.5%	Queen caging + oxalic acid	14.3%	Amitraz	20.0%
Tau-fluvalinate	11.3%	Drone brood removal	14.3%	Monitor monthly and apply miticides when above 3.0 *Varroa* mites/100 bees	20.0%
Formic acid	10.7%	Monitor monthly and apply miticides when above 3.0 *Varroa* mites/100 bees	14.3%	Oxalic acid	20.0%
Thymol	9.6%	Tau-fluvalinate	14.3%	No bee colony import	13.3%
Coumaphos	7.3%	Coumaphos	14.3%	Adult bees mite infestation < 6%	6.7%
Amitraz	5.1%	Formic acid	14.3%	Apply miticides when above *Varroa* 4.0 mites/100 bees	6.7%
Flumethrine	5.1%	Apply miticides when above 2.0 *Varroa* mites/100 bees	14.3%	Brood removal + oxalic acid	6.7%
Queen caging + oxalic acid	5.1%			Queen caging + oxalic acid	6.7%
Drone brood removal	2.8%				
Hop acids	2.3%				
VarroMed	2.3%				
Formic acid + oxalic acid **	1.7%				
Hyperthermia	1.7%				
Trapping comb + oxalic acid	1.1%				
Other	12.4%				

* Total % do not add up to 100% due to rounding to one decimal place. ** applied separately.

**Table 4:** AFB: the positive and negative impact of practice records on honey bee colonies. n = number of practice records.

Positive				Negative	
Complete or clinical recovery *		Number of spores		Residues in honey *	
n = 55		n = 13		n = 3	
Shook swarm	29.1%	Shook swarm	69.2%	Oxytetracycline	33.3%
Tylosin	21.8%	Requeening with hygienic queens	15.4%	Tylosin	33.3%
Oxytetracycline	14.5%	Low colony densities	7.7%	Erythromycin	33.3%
Cultivation tests from adult bees	5.5%	Propolis	7.7%		
Low colony densities	5.5%				
Burning of clinically infected colonies	3.6%				
Lincomycin	3.6%				
Oral vaccination of queen	3.6%				
Propolis	3.6%				
Requeening with hygienic queens	3.6%				
Cultivation tests from honey	1.8%				
Hygienic behavior	1.8%				
Partial shook swarm + oxytetracycline	1.8%				

* Total % do not add up to 100% due to rounding to one decimal place.

## Data Availability

Data is contained within the article or Supplementary Material.

## Supplementary Materials

The following supporting information can be downloaded at: https://www.mdpi.com/article/10.3390/insects16101025/s1, Table S1: Supplemental Material S1—How science supports honey bees: Identification of research on best practices in beekeeping.
